# Frailty and Echocardiographic Indices of Diastolic Function in Older Adults: A Cross-Sectional Analysis

**DOI:** 10.3390/jcm15124645

**Published:** 2026-06-15

**Authors:** Dimitrios Anagnostou, Georgia Vamvakou, Zoi Kollia, Christos Chitas, Nikolaos Theodorakis, Sofia Kalantzi, Aikaterini Spyridaki, Vassilis Milionis, Michalitsa Christodoulou, Ioanna Nella, Efi Gourzoulidou, Sofia Athinaiou, Gesthimani Triantafylli, Maria Nikolaou

**Affiliations:** 1 Geriatric Outpatient Clinic 65+, Sismanogleio-Amalia Fleming General Hospital, 14 25is Martiou Str., 15127 Melissia, Greece; jimdimitris100@gmail.com (D.A.); gvamva@yahoo.gr (G.V.);; 2 Department of Cardiology, Sismanogleio-Amalia Fleming General Hospital, 14 25is Martiou Str., 15127 Melissia, Greece; 3 School of Medicine, National and Kapodistrian University of Athens, 75 Mikras Asias, 11527 Athens, Greece; 4 Department of Internal Medicine, Sismanogleio-Amalia Fleming General Hospital, 14 25is Martiou Str., 15127 Melissia, Greece; 5 Department of Psychiatry, Sismanogleio-Amalia Fleming General Hospital, 1 Sismanogleiou Str., 15126 Marousi, Greece

**Keywords:** frailty, diastole, fried, CFS, diastolic dysfunction, left atrium

## Abstract

**Background**: Frailty in older adults is linked to adverse cardiovascular outcomes, but its relationship with echocardiographic markers of diastolic function remains unclear. We examined associations between frailty measures and indices of diastolic function in community-dwelling older adults. **Methods**: This cross-sectional study included 537 adults aged ≥65 years from a multidisciplinary outpatient clinic. Frailty was assessed using the Fried phenotype, Clinical Frailty Scale (CFS), gait speed, and handgrip strength. Associations with diastolic indices were analyzed using multivariable regression with sequential adjustment. Sensitivity analysis was performed via matching. **Results**: According to the Fried phenotype, 30.8% of participants were robust, 59.7% pre-frail, and 9.5% frail. Indexed left atrial dimension (LAi) was consistently higher in frail individuals. Frailty was also associated with higher odds of elevated right ventricular systolic pressure (>35 mmHg) in unadjusted analyses. Using the CFS, individuals with a score higher than 3 had significantly higher NT-proBNP levels compared to those with a score of 1–2. Higher gait speed and handgrip strength were associated with more favorable cardiac structure, including smaller left heart chamber sizes, and lower natriuretic peptide levels. **Conclusions**: Frailty was independently associated with structural and functional markers of diastolic dysfunction in older adults, particularly left atrial enlargement (as captured in Fried) and NT-proBNP elevation (as captured in CFS), supporting the integration of frailty assessment into cardiovascular risk evaluation.

## 1. Introduction

Frailty is a clinical state in older adults that describes an increased vulnerability to stressors due to age-related decline in physiological reserve across multiple organ systems [1]. Its exact definition has received intense debate [2], as reflected in the existence of multiple definitions. Frailty has been linked to multiple adverse outcomes including falls, bone fractures, hospitalization, disability, dementia, and death [3,4]. Preventing or reversing it is a key goal of geriatric care. Moreover, frailty is a robust predictor of incident cardiovascular disease (CVD), with evidence from cohort studies and meta-analyses showing significantly increased hazards of major adverse cardiovascular events, myocardial infarction, stroke, and mortality among frail individuals [5]. Beyond overt cardiovascular disease, frailty has been linked to subclinical cardiovascular abnormalities, including atherosclerosis and peripheral vascular disease [6,7], and chronic inflammation has been proposed as a key underlying mechanism [5]. In this context, diastolic dysfunction (DD) may represent an additional manifestation of cardiovascular impairment in frail individuals [8,9]. Accordingly, studying relevant diastolic markers can contribute to elucidating the underlying mechanisms linking frailty with DD and might refine risk stratification in older populations. Indeed, the co-existence of DD with a higher Fried phenotype score has been associated with an increased hazard of major adverse cardiac and cerebrovascular events (MACCEs) over 0.6 years of follow-up in a Japanese cohort [10]. While several studies have explored echocardiographic markers in frail populations (Supplementary Table S1) [6,7,8,9,10,11,12,13], almost all assessed frailty using the Fried phenotype and relied on cross-sectional analyses. Collectively, these studies suggest that frailty is associated with markers of elevated left-sided filling pressures (E/e′), increased left ventricular mass and left atrial size, the latter emerging as the most robust association. In the present study, we aim to contribute to this literature in several ways. First, we additionally assess frailty using the Clinical Frailty Scale (CFS) and functional metrics, i.e., gait speed and handgrip strength. Inclusion of the first two is supported by prior evidence demonstrating their prognostic value and possible relevance to diastolic function. For example, in a HFpEF cohort both measures remained independent predictors of 1-year all-cause death or hospitalization after adjustment for the MAGGIC score [14]. Handgrip strength, in addition to its role within the Fried phenotype, is a core criterion for sarcopenia, a condition that has also been linked to diastolic dysfunction (DD) [15]. Second, we evaluate model fit, and, as a sensitivity analysis, apply non-parametric preprocessing (matching) to reduce reliance on model specifications [16]. Third, we narrow the research question by focusing exclusively on diastolic markers, both functional and structural, thereby enabling a more informed selection of confounders. Nevertheless, we acknowledge that cross-sectional analyses remain inherently susceptible to confounding and therefore require cautious interpretation.

## 2. Materials and Methods

**Figure 1:**
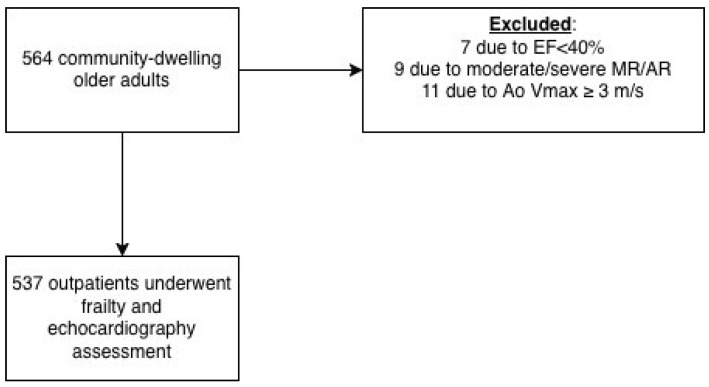
Study flowchart. Abbreviations: MR, mitral regurgitation; AR, aortic regurgitation; Ao Vmax, maximum aortic valve velocity on continuous wave doppler; EF, ejection fraction.

This cross-sectional study was conducted in a multidisciplinary outpatient setting at Amalia Fleming General Hospital in Athens, Greece. It included community-dwelling adults aged ≥65 years assessed between 8 June 2023 and 7 July 2025. Of 564 initially screened participants, those with reduced left ventricular ejection fraction (LVEF < 40%) (*n* = 7), those with moderate-to-severe mitral or aortic regurgitation (*n* = 9) and those with evidence of at least moderate aortic stenosis (*n* = 11) were excluded (Figure 1). These conditions were excluded given their distinct and significant effects on diastolic function. Frailty was assessed using the 5-component Fried phenotype and the Clinical Frailty Scale (CFS). Gait speed was measured over a 4.5-m walk test. Handgrip strength was evaluated using a calibrated hand dynamometer. Participants underwent transthoracic echocardiography, performed by echocardiography-accredited hospital cardiologists. Linear left ventricular dimensions were measured at end-diastole and left atrial dimensions at end-systole in the parasternal long-axis (PLAX) view. Maximum tricuspid regurgitation velocity (TR Vmax) and aortic valve peak velocity (Ao Vmax) were obtained from the echocardiographic view providing the highest measurable velocity. Right ventricular systolic pressure (RVSP) was calculated as 4 × (TR Vmax)^2^ + RAP, where right atrial pressure (RAP) was estimated based on inferior vena cava (IVC) assessment. Body surface area was calculated using the Mosteller formula and used to index echocardiographic measurements. Associations between frailty measures and echocardiographic parameters were examined using univariable and multivariable regression models: linear regression for continuous variables and logistic regression for elevated right ventricular systolic pressure (>35 mmHg). Ordinal regression was used when the H_2_FPEF score was treated as the dependent variable. Sequential models were adjusted for predefined confounders: the base model included age and sex; an intermediate model additionally included body mass index, hypertension, and atrial fibrillation; and the fully adjusted model further included diabetes mellitus, coronary artery disease, hemoglobin concentration and estimated glomerular filtration rate (eGFR). The latter was calculated using the CKD-EPI equation (2021) [17] without inclusion of a race coefficient. Missing data were handled using complete-case analysis. Patterns and proportions of missing data are presented in the Supplementary Material (Supplementary Table S12 and Figure S4). For NT-proBNP, which exhibited a high degree of missingness, multiple imputation [18] was additionally performed under standard missing-data assumptions (MCAR/MAR), yielding similar results (Supplementary Tables S13–S15). For logistic regression models, the extended covariate set was not examined due to the risk of overfitting, in accordance with the events-per-variable (EPV) principle. Model diagnostics were performed using the *performance* R package (version 0.16.0) [19]. For linear models, diagnostic checks included assessment of linearity, homoscedasticity, normality of residuals, influential observations, and multicollinearity. For ordinal regression models, the proportional odds assumption was evaluated using the Brant test. Model diagnostics indicated that log-transformation of NT-proBNP levels and the E/e′ ratio improved model fit.

As part of the HOMER-FRAILTY study, ethical approval was obtained from the local institutional review board (Protocol No. 3975; 7 June 2023). The study was conducted in accordance with the Declaration of Helsinki, and written informed consent was obtained from all participants.

### Sensitivity Analysis

In sensitivity analysis, a preprocessing matching step was implemented using optimal pair matching with the *MatchIt* R package (version 4.7.2) [20], which calls functions from *optmatch* package (version 0.10.8) [21]. Propensity scores estimated using logistic regression were used as the distance measure. One-to-one matching without replacement was performed to estimate the average treatment effect among the treated (ATT), with no caliper restriction applied. Covariate balance in the matched populations is presented in Supplementary Figures S1–S3. Effect sizes were estimated using the g-computation formula using the *marginaleffects* package (version 0.32.0) [22], after fitting regression models that included frailty status, covariates, and their interactions. Cluster-robust variance estimation was used to calculate standard errors, with matching stratum membership specified as the clustering variable. Fried pre-frail and frail categories were contrasted with the robust category, while CFS categories ≥ 3 and ≥4 contrasted with CFS categories 1–2.

## 3. Results

According to the Fried frailty phenotype, 139 (30.8%) participants were robust, 270 (59.7%) pre-frail, and 43 (9.5%) were frail. Using the Clinical Frailty Scale, 263 (58.7%) were in categories 1–2, 133 (29.7%) in category 3, and 52 (11.6%) in more advanced categories. Mean gait speed was 1.09 ± 0.27 m/s in men and 1.00 ± 0.25 m/s in women. Mean grip strength was 27.9 ± 6.68 kg in men and 18.3 ± 4.45 kg in women. Population baseline characteristics are presented in Table 1. Associations that persisted after adjustment are reported in Table 2 and Table 3. Additional associations are provided in Supplementary Tables S4–S8. Considering the intermediate confounder adjustment set, the following associations were observed. Using the Fried phenotype, both prefrail and frail patients had larger left atrial sizes compared with robust individuals. Specifically, prefrail patients had a 0.7 mm/m^2^ higher indexed left atrial linear dimension (LAi) (95% CI: 0.12–1.28), while frail patients had a 1.11 mm/m^2^ higher value (95% CI: 0.07–2.16) compared with robust patients. Frail patients also had higher odds of having right ventricular systolic pressure (RVSP) > 35 mmHg. Based on the extended confounder adjustment set, patients with a CFS score of 3 exhibited an approximately 9% higher E/e′ ratio (95% CI: 0.3–19%) compared with those with scores of 1–2. They also had on average, a 0.71 mm/m^2^ larger indexed left atrial size (95% CI: 0.08–1.34). In adjusted analyses, evidence for higher NT-proBNP levels emerged only among patients in higher CFS categories (≥4). These patients had on average, two-fold higher NT-proBNP levels (≈96% higher; 95% CI: 11–245%) compared with those in categories 1–2. Finally, higher gait speed and grip strength were associated with smaller left heart chamber sizes, as shown in Supplementary Tables S2 and S3. Specifically, each 1 m/s increase in gait speed was independently associated with a 1.29 mm/m^2^ lower LAi (95% CI: 0.23–2.35) after adjustment for the intermediate covariate set, while each 5 kg increase in grip strength was associated with a modest 0.36 mm/m^2^ lower LAi (95% CI: 0.11–0.61). Faster gait speed was also associated with lower left ventricular mass (~13% smaller for a 1 m/s increase; 95% CI: 4–22%), independently of age and sex. Higher grip strength was associated with lower NT-proBNP levels (~12% lower for a 5 kg increase; 95% CI: 0.1–23%), independently of the intermediate adjustment set.

In the sensitivity analysis using the Fried scale, the association with LAi persisted after matching on both the intermediate and extended covariate sets. In contrast, the association with higher RVSP did not remain statistically significant (Supplementary Table S9). For the Clinical Frailty Scale (CFS), when comparing CFS ≥ 3 versus CFS 1–2, differences in log-transformed NT-proBNP remained significant across both covariate sets, while differences in LAi remained significant only in the intermediate covariate set (Supplementary Table S10). When comparing CFS ≥ 4 versus CFS 1–2, only differences in log-transformed NT-proBNP remained significant in both covariate sets; however, this comparison was based on a reduced matched sample (Supplementary Table S11).

Finally, H_2_FPEF score was calculated for all patients and grouped into low (0–1), intermediate (2–5), and high (6–9) categories. In age- and gender-adjusted ordinal logistic regression, higher frailty severity assessed by the CFS was independently associated with higher H_2_FPEF categories. Compared with participants with CFS 1–2, those with CFS 3 had two-fold higher odds of belonging to a higher H_2_FPEF score category (aOR 2.26, 95% CI 1.39–3.74), while participants with CFS ≥ 4 had approximately three-fold higher odds (aOR 3.12, 95% CI 1.44–6.92). A similar association was demonstrated for the Fried frail category compared with the robust category, but only in unadjusted analysis at this sample size.

## 4. Discussion

Across the Fried and CFS frailty scales, indexed left atrial linear dimension (LAi) emerged as the echocardiographic parameter most consistently associated with frailty. Within the Fried frailty phenotype, this association followed a graded pattern, with progressively larger—yet modest—effect sizes observed when prefrail and frail individuals were compared to robust participants. Importantly, the association persisted after adjustment for multiple confounders related to diastolic function and in sensitivity analysis. Although contemporary assessment of diastolic function emphasizes left atrial volume index (LAVI) rather than linear dimensions, only linear measurements were available in the present study. Nevertheless, larger cross-sectional investigations, including those by Ramonfaurt et al. [6] and Nadruz et al. [7] in the ARIC study, have demonstrated associations between increased LAVI and Fried phenotype, even after extensive multivariable adjustment, supporting the validity of our findings. It is possible that frailty contributes to (indexed) left atrial enlargement, that such cardiac remodeling predisposes to frailty, or that both arise from shared underlying processes not fully accounted for in the analysis. Left atrial enlargement is widely recognized as a marker of chronically elevated left ventricular filling pressures, reflecting a cumulative hemodynamic burden that promotes atrial remodeling. The higher odds of right ventricular systolic pressure (RVSP) > 35 mmHg observed in our study may support the presence of such adverse cardiac hemodynamics in frailty. However, this association was not reproduced in sensitivity analysis, and the corresponding logistic regression results were statistically significant only in unadjusted analyses and when adjusting for the intermediate covariate set in the case of Fried (Supplementary Table S8). Still, prior studies have reported increased pulmonary artery systolic pressure (PASP) as well as higher E/e′ in frailty, although these analyses were not extensively adjusted [8,10,12]. Consistent with our findings, the association with E/e′ also attenuates quickly after adjustment [6]. This may reflect the load dependence and dynamic variability of this parameter in frail populations, or simply confounding. In contrast, left atrial size may serve as a more stable “long-term memory” marker of chronic pressure overload, potentially explaining its more consistent association with frailty. Contrary to prior unadjusted analyses [8,10], we observed a minimal increase in lateral e’ tissue Doppler velocity among pre-frail individuals in adjusted analyses. No difference was evident in unadjusted analysis. Gait speed and grip strength, key components of the Fried phenotype, may partly account for the observed differences in LAi, as lower values in both were linked to larger LAi. However, they do not appear to explain differences in RVSP or E/e′, as no statistically significant associations with these parameters were observed in our sample (Supplementary Tables S2 and S3).

Regarding CFS, its grouping was based on ensuring sufficient sample size per category and on the clinical threshold of 4, which marks the onset of very mild frailty; accordingly, scores ≥ 4 were considered to represent frail individuals. Most of them were classified as CFS 4, while higher categories were sparsely represented [CFS 4: 40; CFS 5: 3; CFS 6: 5; CFS 7: 1; CFS 8: 1 patient(s), respectively]. Using this classification, CFS groups captured a highly heterogeneous patient population. Characteristically, adequate matching in sensitivity analysis, defined as standardized mean differences < 10% across covariates, could not be achieved; therefore, regression adjustment was still employed to account for residual imbalance. Despite the above, CFS ≥ 4 was associated with higher NT-proBNP levels compared to CFS 1–2, a finding that persisted after adjustment and in sensitivity analysis. An adjusted association with LAi was also evident at a CFS level of 3 (compared to 1–2). At CFS ≥ 4 this could not be established at this sample size (CFS ≥ 4: 52 patients), although it was observed in unadjusted analysis. One possible interpretation of the above findings is that CFS captures a more clinically overt or decompensated diastolic phenotype, whereas the Fried phenotype may capture more chronic structural remodeling that can be relatively stable at the time of evaluation. Together with the previously reported prognostic relevance of CFS in HFpEF [14], these findings highlight its importance since it remains a rapidly assessed clinical tool. Of course, such interpretations should be considered hypothesis-generating due to the cross-sectional nature of the data.

Finally, objective physical performance measures may provide additional insight into the frailty–cardiac interaction. Faster gait speed was associated with smaller LA size, lower LV dimensions, and reduced LV mass, while higher handgrip strength was associated with lower left chamber size and NT-proBNP levels. These findings may suggest that physical function is linked to cardiac remodeling. They may also provide a hint that dynapenia, sarcopenia, reduced physical activity and cardiovascular deconditioning coexist with worse diastolic profiles.

Frailty as a construct has been thought to include mitochondrial dysfunction, epigenetic alterations, and oxidative stress as biological mechanisms [23]. Alterations in such mechanisms, affect broader metabolic, stress-response, and neuromuscular pathways which in turn contribute to the clinical phenotypes observed [23]. Many of these processes could be linked to impaired myocardial energetics, cardiomyocyte stiffening, abnormal relaxation, and impaired diastolic dysfunction. The most highlighted example is perhaps that of chronic low-grade inflammation (inflammaging) [23,24]. This overlaps with the pathophysiology diastolic dysfunction and HFpEF, in which aging and comorbidity-driven inflammation promote coronary microvascular endothelial dysfunction, reduced nitric oxide–cyclic GMP–protein kinase G signaling, cardiomyocyte stiffening, and interstitial myocardial fibrosis [25]. This process may increase left ventricular filling pressures, impair active relaxation, and contribute to left atrial remodeling.

The above findings could have potential clinical implications. First, they suggest that frailty assessment could serve as a simple, non-invasive screening tool to identify individuals at higher risk of diastolic dysfunction or heart failure with preserved ejection fraction (HFpEF). In support of the latter, higher frailty severity was associated with higher H_2_FPEF score categories, although the score was calculated across the entire cohort and not restricted to patients presenting with dyspnea. Second, the association between physical performance and cardiac structure naturally generates the question of whether interventions targeting physical function, such as resistance training and mobility programs, might influence cardiac remodeling or diastolic function. Although these findings require confirmation in larger, prospective studies, they support the concept that frailty and cardiovascular aging are closely intertwined. Integrating frailty assessment into cardiovascular evaluation may facilitate earlier identification of cardiovascular dysfunction and help identify potentially modifiable pathways for prevention and intervention in older adults.

## 5. Limitations

Several limitations should be acknowledged. First, the observational design precludes causal inference and remains vulnerable to residual confounding. For instance, no systematic evaluation for the presence of pulmonary disease was performed, and unmeasured pulmonary comorbidity may have influenced both frailty status and echocardiographic findings. In addition, collider bias cannot be excluded. Second, echocardiographic parameters were assessed at a single time point, limiting our ability to evaluate temporal relationships. No formal assessment of interobserver variability of echocardiographic measurements was performed. Third, the number of truly frail patients was relatively small (43 according to the Fried criteria and 52 with CFS ≥ 4). Moreover, diastolic dysfunction was approached primarily through continuous echocardiographic indices, with the exception of RVSP, which was analyzed as a binary variable. This differs from the ASE approach, which relies on the simultaneous assessment of E/e′, e′, LAVI, and other parameters to assign a diastolic dysfunction grade [26]. In addition, LAVI was not available in our dataset, and LAi was used instead. Furthermore, NT-proBNP values exhibited a high degree of missingness, which may have reduced statistical power. However, multiple imputation yielded similar results, supporting the robustness of the findings under MAR/MCAR assumptions. Although no formal adjustment for multiple testing was applied, the analyses were exploratory and the examined echocardiographic parameters represented interrelated markers of diastolic function rather than fully independent comparisons. This approach allowed for greater power to detect true effects compared to strict multiplicity adjustment, at the cost of accepting a higher risk of type I error (false positive findings). Finally, the H_2_FPEF score was calculated for all patients, rather than only for dyspneic patients or those with a high pre-test probability of HFpEF, as originally intended [27].

## 6. Conclusions

In conclusion, frailty was independently associated with structural and possibly functional markers of diastolic dysfunction in older adults, particularly left atrial enlargement (as captured by the Fried phenotype) and NT-proBNP elevation (as captured by the CFS score). These findings highlight frailty as a multidimensional syndrome closely linked to cardiac aging and remodeling and support the integration of frailty assessment into cardiovascular risk evaluation. Future studies are needed to determine whether targeting frailty may improve cardiac diastolic profiles and related cardiac outcomes.

## Figures and Tables

**Table 1:** Baseline characteristics according to the Fried frailty phenotype.

Characteristic	Robust *n* = 139	Pre-Frail *n* = 270	Frail *n* = 43	*p*-Value
Age (years)	72 (5)	76 (7)	81 (7)	<0.001
Gender				0.7
Male	56 (40%)	119 (44%)	17 (40%)	
Female	83 (60%)	151 (56%)	26 (60%)	
Weight (kg)	79 (17)	78 (16)	74 (14)	0.2
BMI (kg/m^2^)	28.7 (4.9)	29.4 (5.6)	28.9 (5.7)	0.5
Hypertension	81 (58%)	164 (61%)	35 (81%)	0.020
CAD *	12 (8.6%)	40 (15%)	9 (21%)	0.072
Atrial Fibrillation	8 (5.8%)	40 (15%)	6 (14%)	0.025
Diabetes Mellitus	28 (20%)	80 (30%)	17 (40%)	0.026
eGFR (mL/kg/m^2^)	83 (15)	75 (19)	65 (20)	<0.001
eGFR < 60 mL/kg/m^2^	9 (6.9%)	52 (21%)	12 (32%)	<0.001
Hemoglobin (g/dL)	13.91 (1.36)	13.64 (1.46)	13.11 (1.68)	0.017
Anemia	7 (5.4%)	33 (14%)	12 (32%)	<0.001
MAP (mmHg)	102 (12)	99 (12)	96 (12)	0.017
Heart Rate	71 (11)	70 (11)	74 (13)	0.15
EF				0.2
40–49%	2 (1.4%)	13 (4.9%)	1 (2.4%)	
≥50%	136 (99%)	255 (95%)	41 (98%)	

Continuous variables are presented as mean (SD) and categorical variables as n (%). *p*-values were calculated using the Kruskal–Wallis rank-sum test, Pearson’s chi-squared test, or Fisher’s exact test, as appropriate. * Coronary Artery Disease (CAD) was defined as stenosis of at least 50% on coronary angiography or a history of previous percutaneous coronary intervention and/or coronary aortic bypass graft.

**Table 2:** Diastolic markers explained by the Fried frailty category.

Echo Variable	Fried	Adjustments	β (Difference)	95% CI	95% CI	*p*-Value
e′ (cm/s)	Prefrail	age, gender	0.65	0.13	1.16	0.015
e′ (cm/s)	Prefrail	age, gender, bmi, htn, af	0.56	0.04	1.07	0.034
LAi (mm/m^2^)	Prefrail	-	1.33	0.69	1.97	<0.001
LAi (mm/m^2^)	Frail	-	2.73	1.62	3.84	<0.001
LAi (mm/m^2^)	Prefrail	age, gender	0.69	0.07	1.30	0.029
LAi (mm/m^2^)	Prefrail	age, gender, bmi, htn, af	0.70	0.12	1.28	0.018
LAi (mm/m^2^)	Frail	age, gender, bmi, htn, af	1.11	0.07	2.16	0.037
LAi (mm/m^2^)	Prefrail	age, gender, bmi, htn, af, dm, cad, egfr, hgb	0.63	0.03	1.24	0.041
			**OR**			
RVSP > 35 mmHg	Frail	-	5.87	1.98	18.59	0.002
RVSP > 35 mmHg	Frail	age, gender, bmi, htn, af	3.87	1.04	15.53	0.047

Effects are presented as β coefficients with 95% confidence intervals (CIs) derived from linear regression models. For RVSP > 35 mmHg, odds ratios (OR) with 95% CIs are reported, derived from logistic regression models. The reference group is the robust Fried category.

**Table 3:** Diastolic markers explained by the CFS category.

Echo Variable	CFS	Adjustments	β (Difference)	95% CI	95% CI	*p*-Value
LAi (mm/m^2^)	3	-	1.39	0.74	2.04	<0.001
LAi (mm/m^2^)	4+	-	2.26	1.32	3.20	<0.001
LAi (mm/m^2^)	3	age, gender, bmi, htn, af	0.77	0.16	1.37	0.013
LAi (mm/m^2^)	3	age, gender, bmi, htn, af, dm, cad, egfr, hgb	0.71	0.08	1.34	0.027
			**% difference**			
E/e′	3	-	13	4	22	0.003
E/e′	4+	-	20	7	35	0.002
E/e′	3	age, gender	9	0.3	18	0.043
E/e′	3	age, gender, bmi, htn, af, dm, cad, egfr, hgb	9	0.3	19	0.043
NT-pro BNP (pg/mL)	3	-	55	9	122	0.016
NT-pro BNP (pg/mL)	4+	-	248	93	527	<0.001
NT-pro BNP (pg/mL)	4+	age, gender	102	13	261	0.018
NT-pro BNP (pg/mL)	4+	age, gender, bmi, htn, af	97	13	246	0.018
NT-pro BNP (pg/mL)	4+	age, gender, bmi, htn, af, dm, cad, egfr, hgb	96	11	245	0.020
			**OR**			
RVSP > 35 mmHg	4+	-	4.77	1.99	11.23	<0.001

Effects are presented as β coefficients with 95% confidence intervals (CIs), derived from linear regression models. For E/e′ and NT-proBNP, log-linear regression models were used and results are expressed as percentage differences with 95% CIs, calculated as (exp(β) − 1) × 100. For RVSP > 35 mmHg, odds ratios (OR) with 95% CIs are reported, derived from logistic regression models. The reference group is CFS 1–2.

## Data Availability

Data are available from the corresponding author upon reasonable request, subject to institutional and privacy restrictions.

## Supplementary Materials

The following supporting information can be downloaded at https://www.mdpi.com/article/10.3390/jcm15124645/s1. Table S1: Studies examining the association between frailty and echocardiographic markers; Table S2: Diastolic markers explained by gait speed; Table S3: Diastolic markers explained by handgrip strength. Effect per 5 kg increase in strength; Table S4: Diastolic markers explained by the Fried frailty category; Table S5: Diastolic markers explained by the CFS category; Table S6: Diastolic markers explained by gait speed; Table S7: Diastolic markers explained by handgrip strength. Effect per 1 kg increase in strength; Table S8: Increased TR Vmax and RVSP explained by frailty measures; Table S9: Effects of Fried pre-fral/frail on diastolic indices after matching; Table S10: Effects of CFS ≥ 3 on diastolic indices after matching; Table S11: Effects of CFS ≥ 4 on diastolic indices after matching; Table S12: Missing data percentages; Table S13: NT-proB NP missingess across study variables; Table S14: Log(NT-proBNP) explained by CFS category across the imputed datasets; Table S15: Log(NT-proBNP) explained by CFS category via complete-case analysis (for comparison); Figure S1: Covariate for Fried (Prefrail and Frail) vs. Robust; Figure S2: Covariate balance for CFS ≥ 3 vs. 1–2; Figure S3: Covariate balance for CFS ≥ 4 vs. 1–2; Figure S4: Distribution and patterns of missing data across study variables; Figure S5: Distribution of observed and imputed log-transformed NT-proBNP values across multiply imputed datasets. References [6,7,8,9,10,11,12,13,16,17,21,22] are cited in the supplementary materials.

## Abbreviations

AF, atrial fibrillation; BMI, body mass index; CAD, coronary artery disease; DM, diabetes mellitus; EF, ejection fraction; e′, (lateral) tissue Doppler early diastolic velocity; E/e′, ratio of early mitral inflow velocity to lateral tissue Doppler early diastolic velocity; eGFR, estimated glomerular filtration rate; HFpEF, heart failure with preserved ejection fraction; Hgb, haemoglobin; HTN, hypertension; LA, left atrial linear dimension; LAi, LA indexed to body surface area; LAVI, left atrial volume index; MAP, mean arterial pressure; MCAR, missing completely at random; MAR, missing at random; NT-proBNP, N-terminal pro–B-type natriuretic peptide; PW, posterior wall thickness; RVSP, right ventricular systolic pressure.
