# Supplementary material for: Frailty and Echocardiographic Indices of Diastolic Function in Older Adults: A Cross-Sectional Analysis
Source: J Clin Med. 2026 Jun 15;15(12):4645. doi: 10.3390/jcm15124645 (PMC13302609; doi:10.3390/jcm15124645)
Supplement: Supplementary file 1 [file jcm-15-04645-s001.zip › jcm-4333952-supplementary.pdf]

<u>Supplementary Material</u>

Table of Contents

Literature Review Table .... - 1 -
Results (Gait Speed, Grip Strength) .... - 4 -
Extended Results .... - 6 -
Sensitivity Analysis .... - 10 -
Missing Data .... - 14 -
References .... - 16 -

Literature Review Table

Table S1. Studies examining the association between frailty and echocardiographic markers

Author(s), Year	Number	Analysis	Frailty Tool	Echo Parameters+	Main Findings	Adjustment
Wawrzyniak et al., 2026 (ancillary analysis of FRAPICA) [1]	205 hospitalized patients (mean age 72.9 ± 5.1 years) in a Cardiology department	Cross-sectional	Fried	EF, IVS, LVEDD, PW, LV mass, LVMI, RWT	Frailty associated with higher IVS, LV mass, LMVI, RWT in unadjusted analysis.<br><br>RWT remained significant in all models.	Model 1: age, sex<br><br>Model 2: + weight<br><br>Model 3: + pulmonary disease
Betancourt et al., 2025 [2]	269 older adults (mean age 68.4 ± 6.2 years)	Cross-sectional	Fried	LVEDD, LVEDV, LVESV, RWT, LAVI, RAVI, septal/lateral e’,	Frailty was associated with an increase in LAVI, RAVI, E/e’, PASP, RWT and TR velocity and a decrease in	No adjustment*

				E/A, E/e', TR velocity, PASP	septal and lateral e' velocities; differences in other parameters were minimal despite statistical significance.	
Ramonfaur et al., 2023 (ARIC) [3]	2574 community-dwelling adults (mean age 74 ± 4.4 years)	Cross-sectional (with longitudinal subsection)	Fried	LVMI, LVEDD, wall thickness, LAVI, E/e'	Frailty was associated with increased LVMI, E/e', and LAVI after basic adjustment<br><br>Only LAVI remained significant after extended adjustment.	Basic: age, sex, race, field center<br><br>Extended: + BP, HR, HTN, DM, BMI, eGFR, CHD, MI, stroke
Nadruz et al., 2016 (ARIC) [4]	3991 community-dwelling adults (mean age 75.6 ± 5 years)	Cross-sectional	Fried	LV hypertrophy, LVMI, EF, GLS, E/e', LAVI	Frailty associated with lower GLS, higher LVMI, LV hypertrophy, higher E/e', LAVI, in both unadjusted and adjusted analysis. Difference in EF was minimal despite statistical significance.	age, sex, race, field center
Gharacholou et al, 2015 [5]	247 patients undergoing clinically indicated cardiac ultrasonography exam	Cross-sectional	Fried, gait speed, handgrip strength	LA volume, LV stroke volume, PASP, mitral annular calcification	Frail patients had higher LA volume, PASP, were more likely to have mitral annular calcifications and had lower LV stroke volumes in unadjusted analysis.<br><br>LA volume, LV stroke volume and PASP remained significant in the multivariable model.<br><br>Gait speed had unadjusted correlations with LA	Multivariable model had frailty as the dependent variable. It included age, prior myocardial infaction, heart failure, mitral annular calcification, LA volume, LV stroke volume and PASP as independent variables.

					volume, LV stroke volume, E/e' (medial), PASP, mitral annular calcification presence<br><br>Handgrip strength had unadjusted correlations with LVMI, LA volume, cardiac index PASP.	
Newman et al., 2001 (CHS) [6]	3690 community-dwelling older adults (mean age 71.5 ± 4.6 years) (2461 for LV mass)	Cross-sectional	Fried	LV mass, wall thickness, RWT	Increased wall thickness, RWT, and LV mass were associated with higher odds of frailty.	age, sex, race
Xi et al., 2023 [7]	350 older adults (mean age 70.46 ± 4.72) undergoing pre-operative echo assesment before non-cardiac surgery	Cross-sectional	Fried	IVS, SV, LVMI, LA conduit strain, RV free wall strain, RV 4-chamber strain, LVGLS	Frailty was associated with reduced LA conduit strain and right ventricular free wall strain; differences in other parameters were minimal despite statistical significance.	No adjustment**
Kusunose et al., 2018 [8]	216 patients (mean age 72 ± 4.6) with at least one cardiovascular risk factor	Survival Analysis	Fried	LAVI, SVi, e', E/e', PASP, severe DD	In unadjusted analysis (baseline table) frailty was associated with higher LAVI, E/e', PASP, and lower SVi, e'. Severe DD prevalence also differed between frailty groups.	No adjustment

+: Variables with statistically significant differences across frailty categories.

*: Frailty was associated with the presence of overall diastolic dysfunction (DD), defined according to the 2016 ASE guidelines [13], after adjustment for age, HTN, DM, CHD, COPD, and CKD (adjusted odds ratio [aOR] 3.5; 95% confidence interval [CI]: 1.9–6.4).

**: Frailty was associated with DD after adjustment for sex, HTN, and atrial fibrillation (AF) (aOR 1.5; 95% CI: 1.02–2.2).

Abbreviations: LV, left ventricle; RV, right ventricle; LA, left atrium; LVGLS, left ventricular global longitudinal strain; IVS, interventricular septum; LVEDD, left ventricular end-diastolic diameter; LVEDV, left ventricular end-diastolic volume; LVESV, left ventricular end-systolic volume; LVMI, left ventricular mass index; RWT, relative wall thickness; LAVI, left atrial volume index; RAVI, right atrial volume index; e′, early diastolic mitral annular velocity; E/A, ratio of early (E) to late (A) ventricular filling velocities; E/e′, ratio of early mitral inflow velocity to annular velocity; TR velocity, tricuspid regurgitation velocity; PASP, pulmonary artery systolic pressure; SVi, LV stroke volume indexed; BP, blood pressure; HR, heart rate; HTN, hypertension; DM, diabetes mellitus; BMI, body mass index; DD, diastolic dysfunction; eGFR, estimated glomerular filtration rate; CHD, coronary heart disease; MI, myocardial infarction; ARIC, Atherosclerosis Risk in Communities Study; CHS, Cardiovascular Health Study; FRAPICA, The frailty syndrome in daily practice of interventional cardiology ward.

Results (Gait Speed, Grip Strength)

Table S2. Diastolic markers explained by gait speed

Echo Variable	Adjustments	β (difference)	95% CI	95% CI	P-value
LA (mm)	-	-3.86	-5.77	-1.94	<0.001
LA (mm)	age,gender	-3.65	-5.72	-1.58	0.001
LAi (mm/m^2)	-	-2.94	-3.98	-1.91	<0.001
LAi (mm/m^2)	age,gender,bmi,htn,af	-1.29	-2.35	-0.23	0.018
LAi (mm/m^2)	age,gender,bmi,htn,af,dm,cad,egfr,hgb	-1.18	-2.30	-0.05	0.040
LVEDD (mm)	age,gender	-3.11	-5.07	-1.14	0.002
LVEDDi (mm/m^2)	-	-1.53	-2.60	-0.47	0.005
LVEDDi (mm/m^2)	age,gender,bmi,htn,af	-1.49	-2.58	-0.41	0.007
LVEDDi (mm/m^2)	age,gender,bmi,htn,af,dm,cad,egfr,hgb	-1.35	-2.46	-0.25	0.016
		**% difference**			
LV mass (g)	age,gender	-13	-22	-4	0.006

Effects are presented as β coefficients with 95% confidence intervals (CIs), derived from linear regression models. For LV mass, log-linear regression models were used and results are expressed as percentage differences (% difference) with 95% CIs, calculated as (exp(β)-1)x100.

Table S3. Diastolic markers explained by handgrip strength. Effect per 5 kg increase in strength.

Echo Variable	Adjustments	β (difference)	95% CI	95% CI	P-value
LAi (mm/m^2)	-	-0.52	-0.71	-0.33	<0.001
LAi (mm/m^2)	age,gender	-0.34	-0.61	-0.08	0.012
LAi (mm/m^2)	age,gender,bmi,htn,af	-0.36	-0.61	-0.11	0.004
LVEDDi (mm/m^2)	-	-0.53	-0.71	-0.35	<0.001
LVEDDi (mm/m^2)	age,gender	-0.49	-0.76	-0.21	0.001
LVEDDi (mm/m^2)	age,gender,bmi,htn,af	-0.51	-0.77	-0.26	<0.001
LVEDDi (mm/m^2)	age,gender,bmi,htn,af,dm,cad,egfr,hgb	-0.46	-0.71	-0.20	<0.001
		**% difference**			
NT-pro BNP (pg/mL)	-	-18	-26	-10	<0.001
NT-pro BNP (pg/mL)	age,gender,bmi,htn,af	-12	-23	-0.1	0.048

Effects are presented as β coefficients with 95% confidence intervals (CIs), derived from linear regression models. For NT-proBNP, log-linear regression models were used and results are expressed as percentage differences (% difference) with 95% CIs, calculated as (exp(β)-1)x100.

Extended Results

Table S4. Diastolic markers explained by Fried frailty category

Echo Variable	Category	Adjustments	Effect (β)	95% CI	95% CI	P-value
Log(E/e’)	Frail	-	0.15	0.02	0.29	0.028
e’ (cm/s)	Prefrail	age,gender	0.65	0.13	1.16	0.015
e’ (cm/s)	Prefrail	age,gender, bmi,htn,af	0.56	0.04	1.07	0.034
LA (mm)	Prefrail	-	1.82	0.63	3.01	0.003
LA (mm)	Frail	-	3.00	0.96	5.05	0.004
LA (mm)	Prefrail	age,gender	1.22	0.05	2.39	0.041
LAi (mm/m^2)	Prefrail	-	1.33	0.69	1.97	<0.001
LAi (mm/m^2)	Frail	-	2.73	1.62	3.84	<0.001
LAi (mm/m^2)	Prefrail	age,gender	0.69	0.07	1.30	0.029
LAi (mm/m^2)	Prefrail	age,gender, bmi,htn,af	0.70	0.12	1.28	0.018
LAi (mm/m^2)	Frail	age,gender, bmi,htn,af	1.11	0.07	2.16	0.037
LAi (mm/m^2)	Prefrail	age,gender, bmi,htn,af, dm, cad, egfr, hgb	0.63	0.03	1.24	0.041
Log[LVMI (g/m^2)]	Prefrail	-	0.06	0.01	0.12	0.027
IVS (mm)	Prefrail	-	0.34	0.03	0.66	0.033
LVEDDi (mm/m^2)	Frail	-	1.39	0.25	2.53	0.017
Log [NT-pro BNP (pg/mL)]	Prefrail	-	0.48	0.13	0.83	0.008
Log [NT-pro BNP (pg/mL)]	Frail	-	0.98	0.27	1.70	0.007

Effects are presented as β coefficients witrh 95% confidence intervals (CIs) derived from linear regression models. The reference group is the robust Fried category.

Table S5. Diastolic markers explained by CFS category

Echo Variable	CFS Category	Adjustments	Effect (β)	95% CI	95% CI	P-value
Log(E/e’)	3	-	0.12	0.04	0.20	0.003
Log(E/e’)	4+	-	0.18	0.07	0.30	0.002
Log(E/e’)	3	age,gender	0.09	0.003	0.17	0.043
Log(E/e’)	3	age,gender,bmi,htn,af,dm,cad,egfr,hgb	0.09	0.003	0.18	0.043
LA (mm)	3	-	2.12	0.92	3.32	0.001
LA (mm)	4+	-	2.98	1.27	4.69	0.001
LA (mm)	3	age,gender	1.75	0.55	2.95	0.004
LA (mm)	4+	age,gender	2.14	0.30	3.98	0.023
LAi (mm/m^2)	3	-	1.39	0.74	2.04	<0.001
LAi (mm/m^2)	4+	-	2.26	1.32	3.20	<0.001
LAi (mm/m^2)	3	age,gender,bmi,htn,af	0.77	0.16	1.37	0.013
LAi (mm/m^2)	3	age,gender,bmi,htn,af,dm,cad,egfr,hgb	0.71	0.08	1.34	0.027
Log[LV mass (g)]	3	-	0.06	0.002	0.12	0.043
Log[LVMI (g/m^2)]	3	-	0.07	0.02	0.13	0.010
Log[LVMI (g/m^2)]	4+	-	0.12	0.04	0.21	0.002
LVEDDi (mm/m^2)	4+	-	1.35	0.38	2.32	0.026
IVS (mm)	3	-	0.39	0.08	0.71	0.016
IVS (mm)	4+	-	0.51	0.05	0.96	0.030
LVEDDi (mm/m^2)	4+	-	1.35	0.38	2.32	0.006
PW (mm)	3	-	0.65	0.20	1.10	0.004
PW (mm)	3	age,gender	0.62	0.15	1.10	0.004
PW (mm)	3	age,gender,bmi,htn,af	0.56	0.09	1.04	0.021
Log [NT-pro BNP (pg/mL)]	3	-	0.44	0.08	0.80	0.16
Log [NT-pro BNP (pg/mL)]	4+	-	1.25	0.66	1.84	<0.001
Log [NT-pro BNP (pg/mL)]	4+	age,gender	0.70	0.12	1.28	0.018
Log [NT-pro BNP (pg/mL)]	4+	age,gender,bmi,htn,af	0.68	0.12	1.24	0.018

Log [NT-pro BNP (pg/mL)]	4+	age,gender,bmi,htn,af,dm,cad,egfr,hgb	0.67	0.11	1.24	0.020

Effects are presented as β coefficients witrh 95% confidence intervals (CIs) derived from linear regression models. The reference group is the CFS 1-2 category.

Table S6. Diastolic markers explained by gait speed

Echo Variable	Adjustments	Effect (β)	95% CI	95% CI	P-value
Log(E/e')	-	-0.20	-0.33	-0.07	0.003
e' (cm/s)	-	0.91	0.09	1.74	0.031
LA (mm)	-	-3.86	-5.77	-1.94	<0.001
LA (mm)	age,gender	-3.65	-5.72	-1.58	0.001
LAi (mm/m^2)	-	-2.94	-3.98	-1.91	<0.001
LAi (mm/m^2)	age,gender,bmi,htn,af	-1.29	-2.35	-0.23	0.018
LAi (mm/m^2)	age,gender,bmi,htn,af,dm,cad,egfr,hgb	-1.18	-2.30	-0.05	0.040
Log[LV mass (g)]	age,gender	-0.14	-0.25	-0.04	0.006
Log[LVMI (g/m^2)]	-	-0.12	-0.21	-0.04	0.005
LVEDD (mm)	age,gender	-3.11	-5.07	-1.14	0.002
LVEDDi (mm/m^2)	-	-1.53	-2.60	-0.47	0.005
LVEDDi (mm/m^2)	age,gender,bmi,htn,af	-1.49	-2.58	-0.41	0.007
LVEDDi (mm/m^2)	age,gender,bmi,htn,af,dm,cad,egfr,hgb	-1.35	-2.46	-0.25	0.016
Log [NT-pro BNP (pg/mL)]	-	-0.98	-1.51	-0.44	<0.001

Effects are presented as β coefficients witrh 95% confidence intervals (CIs) derived from linear regression models.

Table S7. Diastolic markers explained by handgrip strength. Effect per 1 kg increase in strength.

Echo Variable	Adjustments	Effect (β)	95% CI	95% CI	Adj. P-value
Log(E/e’)	-	-0.009	-0.014	-0.005	<0.001
e’ (cm/s)	-	0.03	0.005	0.06	0.023
LA (mm)	-	0.10	0.03	0.17	0.004
LAi (mm/m^2)	-	-0.10	-0.14	-0.07	<0.001
LAi (mm/m^2)	age,gender	-0.07	-0.12	-0.02	0.012
LAi (mm/m^2)	age,gender,bmi,htn,af	-0.07	-0.12	-0.02	0.004
Log[LV mass (g)]	-	0.008	0.004	0.011	<0.001
LVEDD (mm)	-	0.18	0.12	0.24	<0.001
LVEDDi (mm/m^2)	-	-0.11	-0.14	-0.07	<0.001
LVEDDi (mm/m^2)	age,gender	-0.10	-0.15	-0.04	0.001
LVEDDi (mm/m^2)	age,gender,bmi,htn,af	-0.10	-0.15	-0.05	<0.001
LVEDDi (mm/m^2)	age,gender,bmi,htn,af,dm,cad,egfr,hgb	-0.09	-0.14	-0.04	<0.001
Log [NT-pro BNP (pg/mL)]	-	-0.04	-0.06	-0.02	<0.001
Log [NT-pro BNP (pg/mL)]	age,gender,bmi,htn,af	-0.03	-0.05	-0.0002	0.048

Effects are presented as β coefficients witrh 95% confidence intervals (CIs) derived from linear regression models.

Table S8. Increased TR Vmax and RVSP explained by frailty measures

Echo Variable	Term	Adjustments	Estimate (OR)	95% CI	95% CI	P-value
TR Vmax > 2.8 m/s	Frail	-	7.03	1.32	52.05	0.028
TR Vmax > 2.8 m/s	CFS 4+	-	5.40	1.24	23.53	0.020
TR Vmax > 2.8 m/s	Gait Speed (m/s)	-	0.07	0.01	0.52	0.009
RVSP>35 mmHg	Frail	-	5.87	1.98	18.59	0.002
RVSP>35 mmHg	Frail	age,gender,bmi,htn,af	3.87	1.04	15.53	0.047
RVSP>35 mmHg	Frail	age,gender,bmi,htn,af,dm,cad,egfr,hgb	6.13	1.44	29.08	0.016
RVSP>35 mmHg	CFS 4+	-	4.77	1.99	11.23	0.000
RVSP>35 mmHg	Gait Speed (m/s)	-	0.26	0.07	0.96	0.042

Effects are presented as odds ratios (OR) witrh 95% confidence intervals (CIs) derived from logistic regression models.

Sensitivity Analysis

Sensitivity analyses were conducted using optimal pair matching implemented with the *MatchIt* R package [9], which calls functions from the *optmatch* package [10]. Optimal matching minimizes the global sum of within-pair distances across the matched sample. Propensity scores estimated using logistic regression were used as the distance measure. Under default settings, one-to-one matching without replacement was performed to estimate the average treatment effect among the treated (ATT), with no caliper restriction applied. Covariate balance before and after matching was evaluated using Love plots and standardized mean differences (SMDs) and is presented in Supplementary Figures 1–2. Absolute SMD values <0.1 were considered indicative of adequate covariate balance after matching. Following matching, effect sizes were estimated using the g-computation formula implemented in the *marginaleffects* package [11], based on regression models including frailty status, covariates, and interaction terms. Cluster-robust variance estimation was used to calculate standard errors, with matching stratum membership specified as the clustering variable. For Fried frailty analyses, pre-frail and frail participants were contrasted with robust participants. For CFS analyses, CFS categories ≥3 and ≥4 were contrasted with CFS categories 1–2. Adequate post-matching covariate balance was achieved in the Fried frailty analyses, with all covariates demonstrating absolute SMD values <0.1 after matching. In contrast, the CFS-based analyses exhibited greater heterogeneity, and adequate balance could not be fully achieved across all covariates.

Fried (Prefrail and Frail) vs Robust

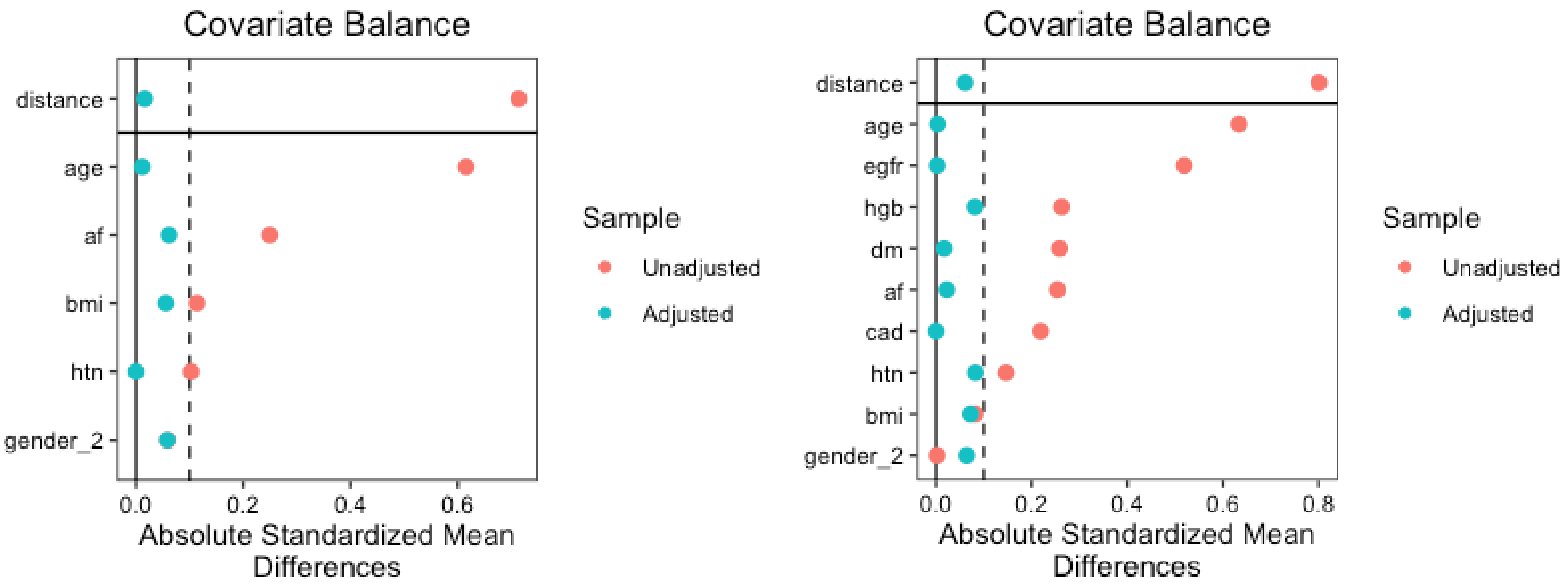

Figure S1. Covariate balance on two mathcing sets. The dashed vertical line indicates a standardized mean difference of 0.1.

Table 9. Effects of Fried pre-fral/frail on diastolic indices after matching

Outcome	ATT	95% CI	P-value	Matching Set	Matched Sample Size
LAi (mm/m^2)	0.88	0.31 - 1.46	0.003	age,gender,bmi,htn,af	138 + 138
LAi (mm/m^2)	0.62	0.06 -1.19	0.032	age,gender,bmi,htn,af,dm,cad,egfr,hgb	126 + 126
e’ (cm/s)	0.65	0.03 - 1.26	0.040	age,gender,bmi,htn,af	138 + 138
e’ (cm/s)	0.60	-0.0125 - 1.22	0.055	age,gender,bmi,htn,af,dm,cad,egfr,hgb	138 + 138

ATT = average treatment effect among the treated (Fried Prefrail & Frail).

CFS ≥ 3 vs 1-2

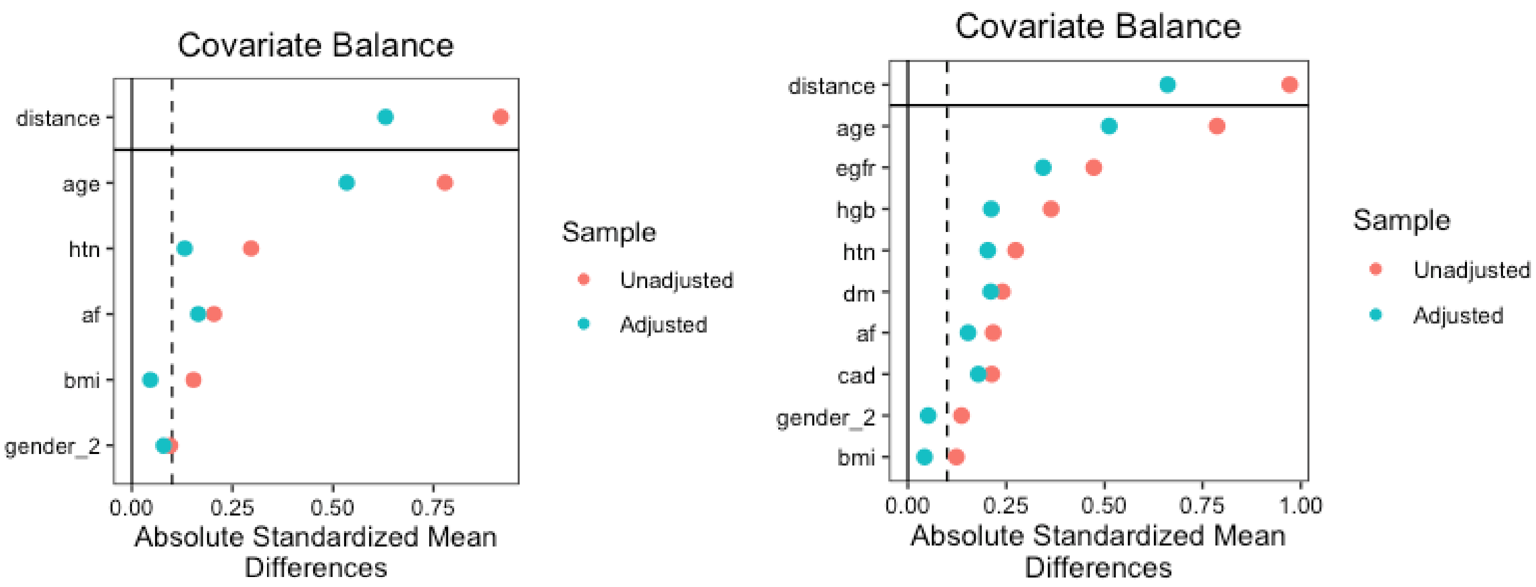

Figure S2. Covariate balance on two mathcing sets. The dashed vertical line indicates a standardized mean difference of 0.1.

Table 10. Effects of CFS ≥3 on diastolic indices after matching

Outcome	ATT	95% CI	P-value	Matching Set	Matched Sample size
Log [NT-pro BNP (pg/mL)]	0.31	0.004 - 0.62	0.047	age,gender,bmi,htn,af	182 + 182
Log [NT-pro BNP (pg/mL)]	0.28	0.008 - 0.56	0.044	age,gender,bmi,htn,af,dm,cad,egfr,hgb	159 + 159
LAi (mm/m^2)	0.86	0.15 - 1.57	0.017	age,gender,bmi,htn,af	182 + 182
LAi (mm/m^2)	0.88	-0.14 - 1.9	0.091	age,gender,bmi,htn,af,dm,cad,egfr,hgb	159 + 159

ATT = average treatment effect among the treated (CFS ≥3)

CFS ≥ 4 vs 1-2

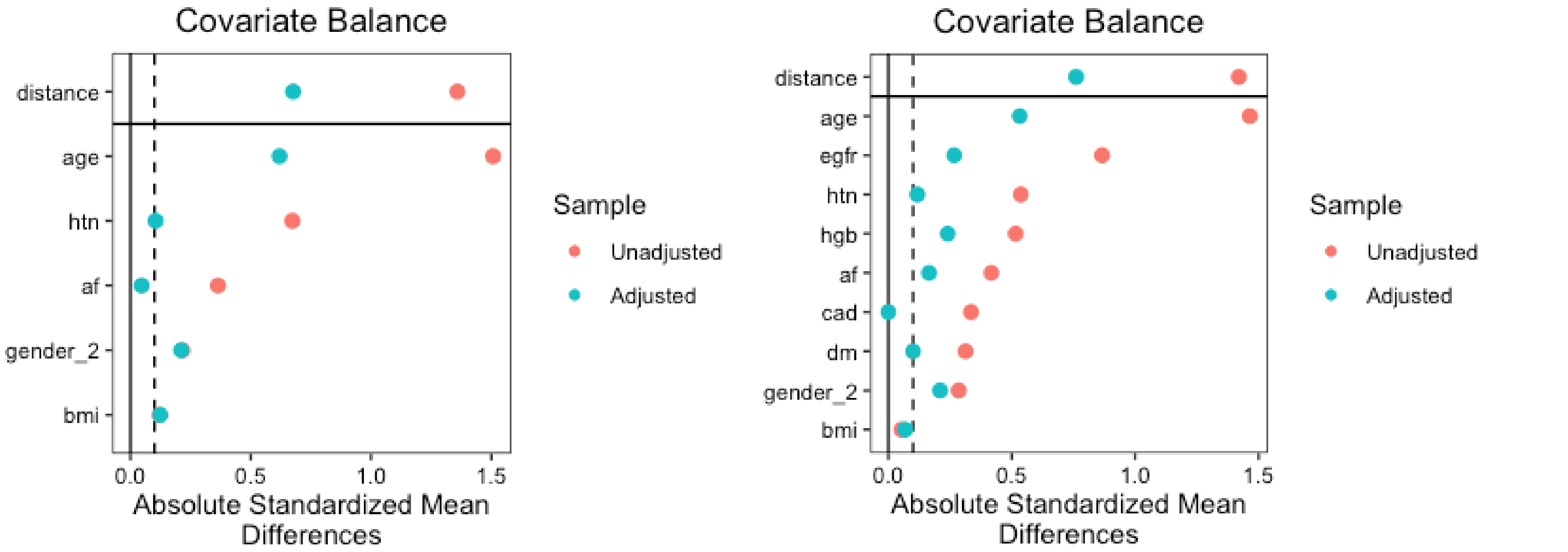

Figure S3. Covariate balance on two mathcing sets. The dashed vertical line indicates a standardized mean difference of 0.1.

Table 11. Effects of CFS ≥4 on diastolic indices after matching

Outcome	ATT	95% CI	P-value	Matching Set	Matched Sample size
Log [NT-pro BNP (pg/mL)]	0.77	0.094 - 1.45	0.026	age,gender,bmi,htn,af	50 + 50
Log [NT-pro BNP (pg/mL)]	0.92	0.282 - 1.57	0.005	age,gender,bmi,htn,af,dm,cad,egfr,hgb	41 + 41

ATT = average treatment effect among the treated (CFS ≥4)

Missing Data

Patterns of missing data are presented in Figure 3 and Table 9. Given the relatively high proportion of missing values for NT-proBNP, Table 10 summarizes the distribution of NT-proBNP missingness across study variables.

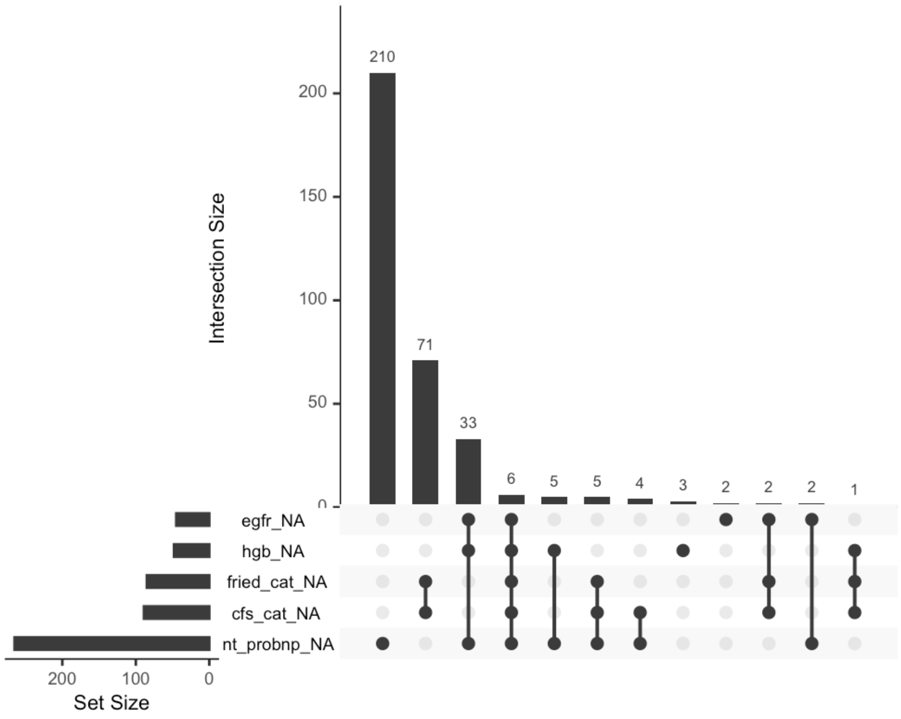

Figure S4. Distribution and patterns of missing data across study variables.

UpSet plot showing the intersection of missingness among selected variables (NT-proBNP, CFS category, FRIED category, hemoglobin, and eGFR). Bars represent the number of individuals for each specific combination of missing variables, while the dot matrix indicates the corresponding pattern of missingness. The most frequent pattern is missing NT-proBNP alone. NA: not available.

Table S12. Missing data percentages

Variable	Missing (%)	Variable	Missing (%)
NT-proBNP	49.35	LA	2.61
CFS category	16.57	BMI	2.42
Fried category	15.83	Grip strength	2.23
Hemoglobin	8.94	IVS	1.86
eGFR	8.38	DM	1.12
E/e’	7.26	Age	0.93
LVMI	4.47	HTN	0.74
LVEDDi	4.28	CAD	0.74
LAi	4.10	RVSP>35	0
Gait speed	3.54	Gender	0
LV mass	2.98	AF	0
LVEDD	2.79		

Table S13. NT-proBNP missingness across study variables

Variable	Not Missing N = 272 (50.7%)	Missing N = 265 (49.3%)	SMD	95% CI
Age	73.69 (6.08)	75.80 (7.17)	-0.32	-0.49, -0.15
Gender			0.07	-0.10, 0.24
Male	112 (41.2%)	118 (44.5%)		
Female	160 (58.8%)	147 (55.5%)		
Weight	76.98 (16.26)	78.26 (15.26)	-0.08	-0.25, 0.09
BMI	28.91 (5.51)	29.19 (5.10)	-0.05	-0.23, 0.12
HTN	160 (59.0%)	166 (63.4%)	-0.09	-0.26, 0.08
CAD	26 (9.6%)	40 (15.3%)	-0.17	-0.34, 0.00
AF	21 (7.7%)	39 (14.7%)	-0.22	-0.39, -0.05
DM	65 (24.0%)	74 (28.5%)	-0.10	-0.27, 0.07
eGFR	79.54 (16.38)	74.91 (19.54)	0.26	0.08, 0.44
eGFR<60	33 (12.3%)	49 (21.9%)	-0.26	-0.43, -0.08
Hgb	13.69 (1.32)	13.63 (1.54)	0.05	-0.13, 0.22
MAP	97.02 (11.37)	101.65 (12.22)	-0.39	-0.57, -0.22
HR	69.07 (9.46)	71.25 (12.21)	-0.20	-0.37, -0.03
Gait speed	1.09 (0.26)	0.98 (0.24)	0.42	0.25, 0.60

Multiple imputation by chained equations (MICE) was applied to handle missing data using the *mice* R package [12]. All variables included in the extended regression models were entered into the imputation model, together with mean arterial pressure (MAP), heart rate (HR), and gait speed as auxiliary variables, not themselves imputed, in order to improve the accuracy and plausibility of the imputations. Predictive mean matching (PMM) was used as the imputation method. Fifty (50) imputed datasets were generated, with twenty (20) iterations per dataset. Statistical analyses were subsequently performed separately within each imputed dataset and the final regression estimates for log(NT-proBNP) were combined across imputations using Rubin's rules. Density plot diagnostics comparing observed and imputed log(NT-proBNP) values are presented in Figure 4. Relevant results for log(NT-proBNP) according to CFS category are presented for the multiply imputed datasets in Table 11 and for the complete-case analysis in Table 12. Findings were generally consistent across approaches.

Table S14. Log(NT-proBNP) explained by CFS category across the imputed datasets

CFS Category	Adjustments	Effect (β)	95% CI	95% CI	P-value
3	-	0.50	0.18	0.82	0.003
4+	-	1.28	0.77	1.80	<0.001
4+	age,gender	0.70	0.17	1.23	0.010
4+	age,gender,bmi,htn,af	0.66	0.14	1.18	0.013
4+	age,gender,bmi,htn,af,dm,cad,egfr,hgb	0.53	0.02	1.04	0.041

Table S15. Log(NT-pro BNP) explained by CFS category via complete-case analysis (for comparison)

CFS Category	Adjustments	Effect (β)	95% CI	95% CI	P-value
3	-	0.44	0.08	0.80	0.16
4+	-	1.25	0.66	1.84	<0.001
4+	age,gender	0.70	0.12	1.28	0.018
4+	age,gender,bmi,htn,af	0.68	0.12	1.24	0.018
4+	age,gender,bmi,htn,af,dm,cad,egfr,hgb	0.67	0.11	1.24	0.020

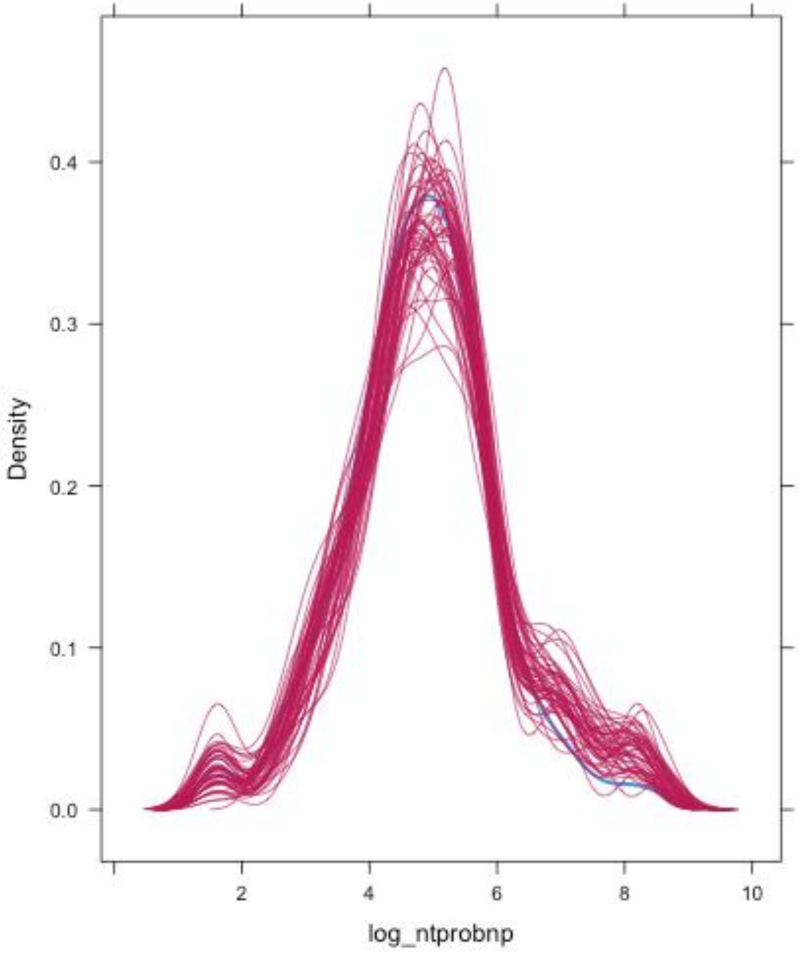

Figure S5. Distribution of observed (blue) and imputed (red) log-transformed NT-proBNP values across multiply imputed datasets.

Abbreviations: AF, atrial fibrillation; BMI, body mass index; CAD, coronary artery disease; CFS, Clinical Frailty Scale; DM, diabetes mellitus; EF, ejection fraction; e′, lateral tissue Doppler early diastolic velocity; E/e′, ratio of early mitral inflow velocity to lateral tissue Doppler early diastolic velocity; eGFR, estimated glomerular filtration rate; Fried, Fried frailty phenotype; HFpEF, heart failure with preserved ejection fraction; Hgb, hemoglobin; HTN, hypertension; IVS, interventricular septum thickness; LA, left atrial linear dimension; LAi, left atrial linear dimension indexed to body surface area; LAVI, left atrial volume index; LVEDD, left ventricular end-diastolic diameter; LVEDDi, left ventricular end-diastolic diameter indexed to body surface area; LV mass, left ventricular mass; LVMI, left ventricular mass index; MAP, mean arterial pressure; MAR, missing at random; MCAR, missing completely at random; NT-proBNP, N-terminal pro–B-type natriuretic peptide; PW, posterior wall thickness; RVSP, right ventricular systolic pressure.

References

1. Wawrzyniak, S.; Wołoszyn-Horák, E.; Cieśla, J.; Schulz, M.; Krawiec, M.; Janik, M.; Wojciechowski, P.; Dajnowska, I.; Szablewska, D.; Bartoszek, J.; et al. The Impact of Frailty on Left Ventricle Mass and Geometry in Elderly Patients with Normal Ejection Fraction: A STROBE-Compliant Cross-Sectional Study. *Journal of Cardiovascular Development and Disease* **2026**, *13*, doi:10.3390/jcdd13010050.
2. Betancourt, D.; Zuluaga, J.; Arango, F.; Murillo, T.; Hincapié, D. Association between Frailty and Echocardiographic Findings in Hospitalized Older Adults with Preserved Ejection Fraction. *Eur Heart J Open* **2025**, *5*, oeaf087, doi:10.1093/ehjopen/oeaf087.
3. Ramonfaur, D.; Skali, H.; Claggett, B.; Windham, B.G.; Palta, P.; Kitzman, D.; Ndumele, C.; Konety, S.; Shah, A.M. Bidirectional Association Between Frailty and Cardiac Structure and Function: The Atherosclerosis Risk in Communities Study. *Journal of the American Heart Association* **2023**, *12*, e029458, doi:10.1161/JAHA.122.029458.
4. Nadruz, W., Jr; Kitzman, D.; Windham, B.G.; Kucharska-Newton, A.; Butler, K.; Palta, P.; Griswold, M.E.; Wagenknecht, L.E.; Heiss, G.; Solomon, S.D.; et al. Cardiovascular Dysfunction and Frailty Among Older Adults in the Community: The ARIC Study. *J Gerontol A Biol Sci Med Sci* **2017**, *72*, 958–964, doi:10.1093/gerona/glw199.
5. Gharacholou, S.M.; Tashiro, T.; Cha, S.S.; Scott, C.G.; Takahashi, P.Y.; Pellikka, P.A. Echocardiographic Indices Associated With Frailty in Adults ≥65 Years. *American Journal of Cardiology* **2015**, *116*, 1591–1595, doi:10.1016/j.amjcard.2015.08.023.
6. Newman, A.B.; Gottdiener, J.S.; Mcburnie, M.A.; Hirsch, C.H.; Kop, W.J.; Tracy, R.; Walston, J.D.; Fried, L.P.; Cardiovascular Health Study Research Group Associations of Subclinical Cardiovascular Disease with Frailty. *J Gerontol A Biol Sci Med Sci* **2001**, *56*, M158-166, doi:10.1093/gerona/56.3.m158.
7. Xi, L.; Xuemei, Z.; Ling, Y.; Changchun, C.; Zhuo, H.; Jinyang, Q.; Xin, W. Correlation between Frailty and Cardiac Structure and Function in Echocardiography in Elderly Patients with Normal Ejection Fraction. *Aging Clin Exp Res* **2023**, *35*, 775–784, doi:10.1007/s40520-023-02363-5.
8. Kusunose, K.; Okushi, Y.; Yamada, H.; Nishio, S.; Torii, Y.; Hirata, Y.; Saijo, Y.; Ise, T.; Yamaguchi, K.; Yagi, S.; et al. Prognostic Value of Frailty and Diastolic Dysfunction in Elderly Patients. *Circulation Journal* **2018**, *82*, 2103–2110, doi:10.1253/circj.CJ-18-0017.

9. Ho, D.; Imai, K.; King, G.; Stuart, E.A. MatchIt: Nonparametric Preprocessing for Parametric Causal Inference. *Journal of Statistical Software* **2011**, *42*, 1–28, doi:10.18637/jss.v042.i08.
10. Hansen, B.B.; Klopfer, S.O. Optimal Full Matching and Related Designs via Network Flows. *Journal of Computational and Graphical Statistics* **2006**, *15*, 609–627, doi:10.1198/106186006X137047.
11. Arel-Bundock, V.; Greifer, N.; Heiss, A. How to Interpret Statistical Models Using Marginaleffects for R and Python. *Journal of Statistical Software* **2024**, *111*, 1–32, doi:10.18637/jss.v111.i09.
12. Buuren, S. van; Groothuis-Oudshoorn, K. Mice: Multivariate Imputation by Chained Equations in R. *Journal of Statistical Software* **2011**, *45*, 1–67, doi:10.18637/jss.v045.i03.
